# Integrated Microfluidic–Electromagnetic System to Probe Single-Cell Magnetotaxis in Microconfinement

**DOI:** 10.3390/bioengineering10091034

**Published:** 2023-09-01

**Authors:** Brianna Bradley, Juan Gomez-Cruz, Carlos Escobedo

**Affiliations:** Department of Chemical Engineering, Queen’s University, Kingston, ON K7L 3N6, Canada

**Keywords:** microfluidics, magnetotactic bacteria, microswimmer, magnetotaxis, single-cell analysis, bacterial taxis

## Abstract

Magnetotactic bacteria have great potential for use in biomedical and environmental applications due to the ability to direct their navigation with a magnetic field. Applying and accurately controlling a magnetic field within a microscopic region during bacterial magnetotaxis studies at the single-cell level is challenging due to bulky microscope components and the inherent curvilinear field lines produced by commonly used bar magnets. In this paper, a system that integrates microfluidics and electromagnetic coils is presented for generating a linear magnetic field within a microenvironment compatible with microfluidics, enabling magnetotaxis analysis of groups or single microorganisms on-chip. The platform, designed and optimised via finite element analysis, is integrated into an inverted fluorescent microscope, enabling visualisation of bacteria at the single-cell level in microfluidic devices. The electromagnetic coils produce a linear magnetic field throughout a central volume where the microfluidic device containing the magnetotactic bacteria is located. The magnetic field, at this central position, can be accurately controlled from 1 to 10 mT, which is suitable for directing the navigation of magnetotactic bacteria. Potential heating of the microfluidic device from the operating coils was evaluated up to 2.5 A, corresponding to a magnetic field of 7.8 mT, for 10 min. The maximum measured heating was 8.4 °C, which enables analysis without altering the magnetotaxis behaviour or the average swimming speed of the bacteria. Altogether, this work provides a design, characterisation and experimental test of an integrated platform that enables the study of individual bacteria confined in microfluidics, under linear and predictable magnetic fields that can be easily and accurately applied and controlled.

## 1. Introduction

Magnetotactic bacteria (MTB) are a unique group of aquatic, flagellated bacteria, characterised by the presence of intracellular ferromagnetic nanoparticle-based organelles called magnetosomes [1]. These organelles consist of an iron-containing crystal, typically magnetite, Fe_3_O_4_, encompassed in a phospholipid bilayer [2]. A single cell has approximately 20 to 50 magnetosomes arranged in one or several chains of various lengths in the cytoplasm, parallel to the axis of motility of the cell [3,4,5]. This colinear arrangement maximises the dipole moment of the magnetic chain, which acts like a compass needle, causing the cell to passively orient then actively swim along external magnetic and geomagnetic field lines, a navigation behaviour called magnetotaxis [4,6]. The navigation of MTB can be effectively and actively directed by applying a magnetic field, enabling their use as biomicrorobots in applications such as targeted drug delivery [7,8,9] and bioremediation [10,11].

MTB respond to the geomagnetic field, which has a magnetic flux density of 25 to 65 μT [12]. Therefore, any externally applied magnetic field with a greater intensity may be used to overcome the geomagnetic field and effectively direct the cells [1]. The interaction with magnetic fields can be influenced by the magnetosome size and purity of the iron content [13]. Recent studies have shown that the fraction of bacteria aligning to the direction of the magnetic field increased from 0 to approximately 90% as the magnetic field was increased from 0 to 1.6 mT, but a threshold value of 1.1 mT was found where the fraction of bacteria aligning to the magnetic field did not increase further [14]. A recent study by Waisbord et. al. used a magnetic field of 1.75 mT to successfully direct MTB in corrugated microchannels [15]. The magnetotaxis response’s dependence on the applied magnetic field has also been studied in two-phase systems. Vincenti et al. studied the vortical flow field motion exerted by MTB in droplets at different magnetic field strengths [16]. They reported a minimum threshold value of 0.4 ± 0.1 mT and an increased angular velocity as the magnetic field increased to 2 mT then 4 mT [16]. Recent technological developments combine spectrometry with electromagnetic coils that enable the measurement of the magnetotactic velocity distribution of bacterial populations (i.e., bulk) in relatively large fluidic environments by an open-source automated, magnetic optical density meter [17]. In terms of combined taxis-driven bacterial navigation, Bennet et al. employed a tri-axial coil system to investigate magneto-aerotaxis, which provided key findings on the influence of the magnetic field orientation and strength on the organisation of bacteria within oxygen gradients which leads to the formation of microaerotactic bands [18].

Applying an external magnetic field to microconfined MTB is challenging. While bar magnets have been commonly used to direct MTB in microfluidic environments [19,20,21,22], their size and inherent curvilinear nature of their magnetic field lines are far from ideal to achieve a detailed characterisation of the cells’ swimming behaviour at the single-cell level [23,24]. Electromagnetic coils, on the other hand, have been rarely used to study magnetotactic behaviour in continuous microfluidic environments due to the difference in length scale between the microscope setup, the microfluidics and the cells. Coils external to the microscope require a large amount of space. Also, components of the microscope, such as protrusions, light sources and acquisition equipment, may interfere with the placement of the coils. Furthermore, a large separation between the coils would provide a weak magnetic field at the centre of the coils, where the microfluidic chip should be positioned to ensure exposure to linear field lines. At this large separation, a higher current must be used to produce a magnetic field strong enough to control the MTB, so the power source and the materials would have to be able to accommodate the electrical requirements. However, coils, when aligned along a common axis, produce linear and uniform magnetic field lines through the central space normal to the planes containing them [25]. The linear magnetic field lines produced by the pair of coils are ideal for directing MTB along a linear pathline and to study the deviation from a linear trajectory due to external factors. Additionally, the intensity of the magnetic field can be precisely controlled by changing the electric current supplied to the coils [25].

Here, we present a platform that provides a precise, controllable and linear magnetic field within a space that is compatible with length scales for the study of individual cells within a region of interest and in flow conditions, through the integration of electromagnetic coils and microfluidics. This platform ensures a uniform application of a precise magnetic field throughout entire microchannels, allowing the detailed study of the behaviour of groups or individual microswimmers in simple or complex microfluidic environments. The platform was designed and optimised through finite element analysis (FEA) to produce a uniform, linear magnetic field within an envelope of 216 mm^3^ where the region of interest for single-cell study is located. The magnetic field produced by the fabricated platform was able to produce precisely controlled fields from 1 to 10 mT, a range compatible with the requirements for magnetotactic MTB studies. Additionally, the temperature of the microfluidic device in the centre is measured to account for the power dissipation of the electromagnetic coils. Finally, the coils are used to produce a magnetic field to direct the navigation of MTB in a microfluidic device.

## 2. Materials and Methods

### 2.1. Overview of the Microfluidic–Electromagnetic Platform for MTB Studies

**Figure 1:**
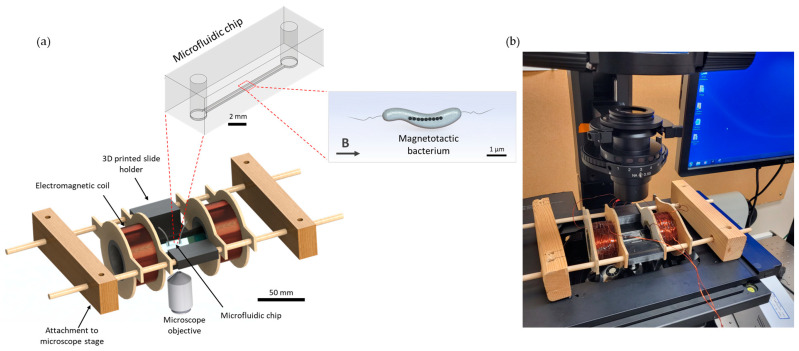
(**a**) A schematic diagram of the microfluidic–electromagnetic coils platform, and (**b**) an actual picture of the device. A microfluidic device can be placed between the two coils in the 3D-printed slide holder, and MTB can be visualised in the microscope during experiments. The platform enables the integration of macroscopic scale electromagnetic coils and holders, a millimeter-scale microfluidic device with microscopic features and the visualisation of micrometer-scale MTB. The two wooden blocks at the ends can be screwed into the microscope stage to ensure stability of the setup.

The platform, with a total dimension of 236 mm, consisted of two coils with a radius of 21 mm, width of 30 mm and a stacked wire thickness of 7.7 mm separated by 51.3 mm and a central area for placing microfluidic chips with complete open access and spatial clearance between coils. A schematic representation and an actual picture of the device are shown in Figure 1. The electromagnetic coils were designed using computer-aided design (CAD) tools, as described below.

The centres of the coil spindles were made with 1.5-inch diameter PVC pipe, and the sides were made from 1/8-inch thick plywood. A hole was cut into the centre of the plywood, and the PVC pipe was inserted between two plywood pieces for each coil and secured in place with epoxy. The plywood was cut such that there were two support structures on either side to be used to suspend the coils. Copper magnetic wire (22 AWG, Digi-Key Electronics, Thief River Falls, MN, USA) was used with 400 turns on each coil. A microscope slide holder that fits standard microscope slides of 25 mm × 75 mm, which also serves as a spacer for the coils, was 3D printed using a stereolithography (SLA) technique (Photon S, ANYCUBIC, Shenzhen, China). Wooden blocks were attached to the microscope platform using screws, and wooden dowels were inserted between the blocks. The coils and microscope slide holder were attached to the wooden dowels such that they were suspended above the microscope objective. The coils could slide along the dowels to adjust the separation if needed. The separation of the coils for this characterisation was 80.95 mm, centre-to-centre. The wires were connected to a 30 V 10 A DC power supply (Sky TopPower, STP3010H, Shenzhen, China). The magnetic field produced by the coils was measured experimentally using a Hall effect gaussmeter (F.W. Bell 5070, F.W. Bell, Portland, Oregon).

### 2.2. Computer-Aided Design and Finite Element Analysis

The components of the microfluidic–electromagnetic coils platform were designed in SolidWorks 2022 (Dassault Systèmes, France) computer-aided design (CAD) software. All parts were assembled within SolidWorks to ensure the correct spacing to accommodate the microscope components.

Finite element analysis (FEA) was used to predict the magnetic field generated from the coils with electric currents ranging from 0.2 to 3.2 A. A 3D model of the coils was generated using the CAD tools in COMSOL Multiphysics 5.6 software (COMSOL, Inc., Sweden). The simulated geometry consisted of two solids of revolution that represent the volume of the wire. A sphere encompassed the coils with the material set to air. A 12 mm × 30 mm × 3 mm rectangular block was centred at the origin between the two coils to represent a microfluidic device. To represent the properties of the polydimethylsiloxane (PDMS) used to fabricate the actual microfluidics, the relative permeability, the relative permittivity and the electrical conductivity were set to 1 [26], 2.69 [27] and 4 × 10^−13^ Sm [28], respectively. An extremely fine physics-controlled mesh was used for all domains. The steady-state Magnetic Fields physics interface was used with Ampère’s Law and Coil Nodes option. The coil nodes were set up with the homogenised multiturn coil model, where the coil represents a bundle of wires separated by an insulator. The circular coil type was selected since the wires are wound in circles around the same axis, and the coil excitation was set to current. The coil wire conductivity was set to that of copper, 5.8 × 10^7^ S/m. The same values as the experimental setup were used for the number of turns and the coil wire cross-section area, which were 400 and 22 AWG, respectively. To compare the magnetic field produced by a permanent, neodymium bar magnet to that of the coils, the magnetic field of a bar magnet was modelled using the COMSOL Magnetic Fields, No Currents physics interface. The geometry consisted of a 20 mm × 50 mm × 10 mm block with the material set to neodymium (solid) surrounded by a 100 mm radius sphere with the material set to air. The recoil permeability of neodymium was inputted as 1.05 [29]. An extremely fine physics-controlled mesh was used for all domains. The Magnetic Flux Conservation node was used for the permanent magnet with the remanent flux density magnetisation model. The remanent flux density norm was set to 1.3 T [30]. The magnetisation of the sphere of air was set to zero, and magnetic insulation was applied to the boundaries of the sphere.

### 2.3. Bacteria

*Magnetospirillum magneticum* strain AMB-1 (ATCC 700264) was used for magnetotaxis experiments. Revised magnetic *Spirillum* growth medium (ATCC medium 1653) was used to grow a liquid culture of *M. magneticum*. The medium consists of 10.0 mL Wolfe’s vitamin solution, 5.0 mL of Wolfe’s mineral solution, 2.0 mL ferric quinate, 0.45 mL resazurin, 0.68 g KH_2_PO_4_, 0.12 g NaNO_3_, 0.035 g ascorbic acid, 0.37 g tartaric acid, 0.37 g succinic acid and 0.05 g sodium acetate in 1.0 L distilled water. NaOH was used to adjust the pH of the medium to 6.75. Prior to experiments, the growth medium was inoculated in a 50% ratio with *M. magneticum* in a volume of 20 mL, and the cells were incubated at 30 °C for 48 h. The vials were closed with minimal headspace to create microaerobic conditions. Resazurin was included as a reduction–oxidation potential indicator, which causes the solution to turn from pink to clear as the cells consume oxygen. Experiments were conducted after the solution turned clear since this change indicates cell growth.

### 2.4. Microfluidic Device Design and Fabrication

While any microfluidic design which fits on a standard microscope slide of 25 mm × 75 mm can be used in the microfluidic–electromagnetic coils platform, a microfluidic device with a 50 μm deep, 300 μm wide straight channel was used to verify the magnetotactic response of MTB in the system. The channel was designed using SolidWorks and fabricated using soft lithography. A mould for the microfluidic device was fabricated using an SLA 3D printer (ANYCUBIC Photon S, China) with 405 nm UV-sensitive resin with 50 μm layer thickness. A monolayer of 1H,1H,2H,2H-perfluorooctyltriethoxysilane (MilliporeSigma, Burlington, MA, USA) was formed on the surface of the mould using vapor deposition to prevent adherence to it. Polydimethylsiloxane (PDMS, SYLGARD 184, Dow Silicones Corporation, Midland, MI, USA) was mixed in a 10:1 *w*/*w* ratio of base and curing agent then degassed for 30 min. The PDMS was then poured into the mould, degassed, then cured at 75 °C for 30 min. The cured PDMS was demoulded, then a biopsy puncher was used to create holes for the inlets and outlets. The surfaces of the PDMS and a glass microscope slide were oxidised via plasma oxidation in a plasma cleaner (Harrick Plasma, Ithaca, NY, USA). The two surfaces were immediately placed in contact to form a Si-O-Si bond. The MTB were introduced to the microfluidic device using a 1 mL syringe. An inverted microscope (Olympus IX83, Hamburg, Germany) with a high-speed CMOS camera (Zyla-4.2-CL10, ANDOR, Belfast, Ireland) was used to record brightfield videos of MTB magnetotaxis in the microfluidic device positioned in the microfluidic–electromagnetic coils platform with a 300 μm × 300 μm field of view and a distance (z-direction) of 15 μm from the bottom of the channel. Videos were recorded at room temperature using 40× magnification (NA 0.6 LUCPlanFL N, Olympus, Shinjuku City, Tokyo, Japan) at 10 FPS. ImageJ 1.54f was used to post-process the videos, and the ImageJ Manual Cell Tracking plugin was used to track the individual cells. The speeds and trajectory angles were estimated using a custom Python code from the tracked positions and timestamps from the ImageJ-processed sequences.

### 2.5. Temperature Measurements

The temperature at the centre of the system was measured using an infrared thermometer (MAXIMUM, Model no. 057-4632-8, Shanghai, China) for applied currents from 0.5 to 2.5 A. The PDMS microfluidic device, as described in the Section 2.4, was placed in the slide holder of the coils, and the temperature of the surface of the PDMS was measured every 30 s for 10 min to determine the temperature increase that the MTB might experience for each current (*n* = 3).

## 3. Results and Discussion

**Figure 2:**
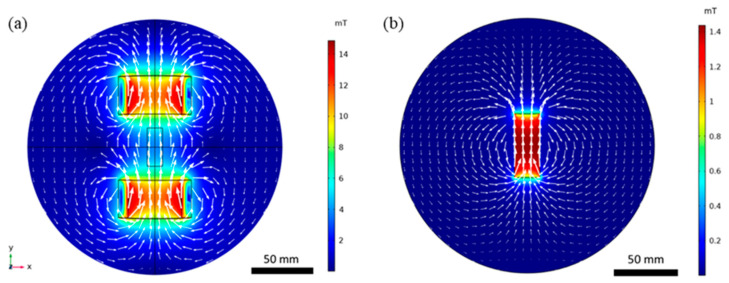
Simulated magnetic flux density of (**a**) a pair of electromagnetic coils with a current of 1.3 A and (**b**) a neodymium bar magnet. White arrows represent the magnitude and direction of the magnetic flux.

The design and redesign of the platform were supported by FEA, in order to find suitable, or the best possible, conditions for MTB magnetotaxis studies within the microfluidic device. FEA was also used to compare the strength and form of the magnetic field lines produced by the designed coils and a neodymium magnet, both commonly used in magnetotaxis-related research. Figure 2 presents the results of the magnetic field simulations using FEA, as described in the Section 2. The magnetic field lines generated by the electromagnetic coils (Figure 2a) and a bar magnet (Figure 2b) were simulated to show the path that the MTB would follow when exposed to the magnetic field. The simulated magnetic field lines of the coils are linear at the centre of the system within a distance of 3 mm, in agreement with previous reports in the literature, with the magnetic field lines looping around one coil to follow a curvilinear trajectory to the other coil [20]. In comparison, the bar magnet fails to produce linear magnetic field lines within a space that can be used to place the microfluidics and, ultimately, study MTB. It produces a linear magnetic field inside the magnet and curvilinear field lines in the medium surrounding the bar. The linear field lines produced in the centre of the coils are ideal, and necessary, to obtain detailed information on the magnetotactic trajectories of MTB, without biasing the pathlines due to a bend in the applied magnetic field. Therefore, it is of critical importance that MTB align to linear field lines and follow a linear path to study their magnetotactic behaviour at the single-bacterium level.

An important parameter which affects the characteristics of the magnetic field produced by the coils is the separation of the two coils. In a traditional Helmholtz coil arrangement, the coils have a separation equal to the radius, and the field in between the coils has a uniform magnitude and direction. For this work, the coils must be placed further apart to accommodate the space for the microfluidics and the microscope objective and condenser. The magnetic flux density along the central axis of two parallel coils, $B_{y}$, is shown in Equation (1), where $\mu_{0}$ is the permeability of free space, R is the coil radius, $N_{coils}$ is the number of turns of the wire and I is the electric current [1].

(1) $$B_{y}\left(y\right)=\frac{\mu_{0}\left(\pi R^{2}N_{coils}I\right)}{4\pi }\left[\frac{1}{{\left(R^{2}+{\left(y+a\right)}^{2}\right)}^{\frac{3}{2}}}+\frac{1}{{\left(R^{2}+{\left(y-a\right)}^{2}\right)}^{\frac{3}{2}}}\right]$$

**Figure 3:**
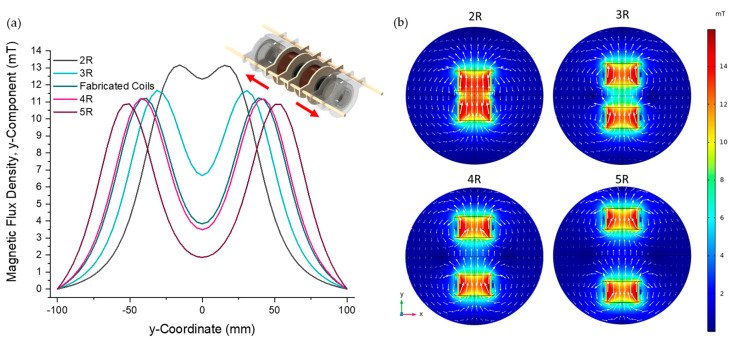
(**a**) Simulated magnetic flux density along the centre axis of the electromagnetic coils for a coil separation from 2R to 5R with a current of 1.3 A. As the separation increases, the intensity of the magnetic flux density decreases at the centre between the coils. Inset: A schematic representation of the microfluidic–electromagnetic coils platform demonstrating the coil separations increasing, represented by the red arrows. (**b**) The simulated magnetic flux densities for coil separations from 2R to 5R on an x–y-plane at the z = 0 position, which intersects the coils symmetrically.

The origin is located at the centre between the two coils, and a is the distance from the origin to each coil. Since $B_{y}$ is inversely proportional to a, the magnetic flux density decreases as the coil separation increases. The simulated magnetic flux density that results from different separations of the coils is shown in Figure 3. As the coils are moved farther apart, the intensity of the magnetic field at the centre decreases. As the coils are moved from a separation of double the radius (2R) to triple, quadruple and quintuple the radius (3R, 4R, 5R), the simulated magnetic field decreases from 12.34 mT to 6.67 mT, 3.49 mT and 1.86 mT, respectively. These findings concur with the equation representing the magnetic flux density along the central axis of two parallel coils shown in Equation (1).

**Figure 4:**
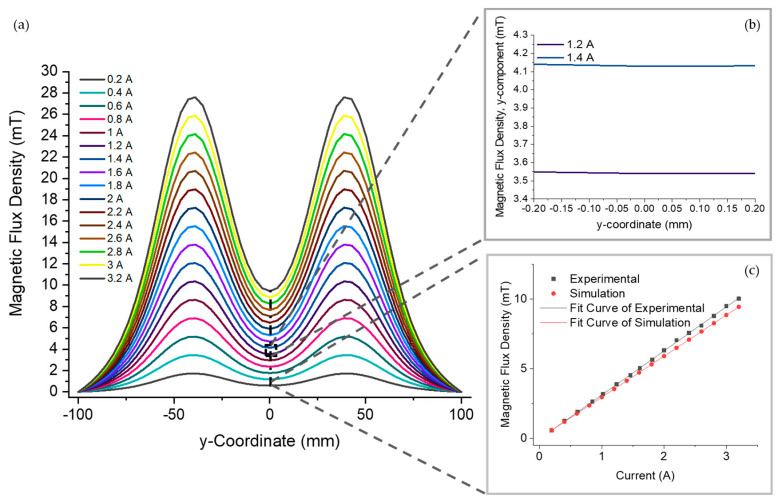
(**a**) Simulated magnetic flux density along the center axis of the electromagnetic coils at different currents with the fabricated coil separation. (**b**) The magnetic flux density at a scale relevant to MTB experiments for 1.2 A and 1.4 A. (**c**) Simulated and experimental magnetic flux density at the centre of the coils for a varied current.

An advantage to electromagnetic coils is that the strength of the magnetic flux density can be changed simply by altering the current supplied to the coils. The effect of changing the electric current on the magnitude of the magnetic flux density was investigated experimentally and through FEA simulations, with an electric current from 0.2 to 3.2 A. In Figure 4a, the coils were simulated with the same separation as the fabricated coils, and the magnetic flux density along the central axis of the coils is plotted for each current. The overall magnetic flux density increases in proportion to the current in agreement with Equation (1). As described in the theory, the magnetic flux density is at the highest intensity at each coil (y=±a), whereas at the centre of the system between the coils (y=0), there is a minimum intensity. However, for a region of interest that is compatible with the microfluidic MTB studies involving multiple cells (Figure 4b), e.g., within 50 µm, the change in magnetic flux density in the y-direction is less than 1 μT, which is insignificant in a magnetotaxis context. Figure 4c shows the magnetic flux density at the centre point between the coils for both simulated and experimental measurements. The coils produced a magnetic flux density greater than 2 mT for currents above 0.61 A, which is the minimum desired value to direct the navigation of MTB. Thus, the microfluidic–electromagnetic coils platform produces a sufficient magnetic flux density for studying MTB magnetotaxis, and the electric current can be varied to study the effect of changing the magnetic flux density on MTB magnetotaxis.

The linear regression of the experimental measurements shows a strong linear correlation between the current and magnetic flux density, with an R^2^ value of 99.97%. When comparing the regression models for the experimental measurements and the simulation results, the intercept values were not statistically different (*p* = 0.091). The statistical difference (*p* < 0.001) between slopes is caused by discrepancies between the fabricated and simulated coils as well as the positioning of the elements to measure the magnetic field, as expected. As shown in Figure 4a, a slight deviation in position from the centre will result in a higher value of the magnetic flux density. At higher currents, the difference in magnetic flux density between the centre position and the off-centre position will be larger, as can be seen from the steeper slopes in the plot.

**Figure 5:**
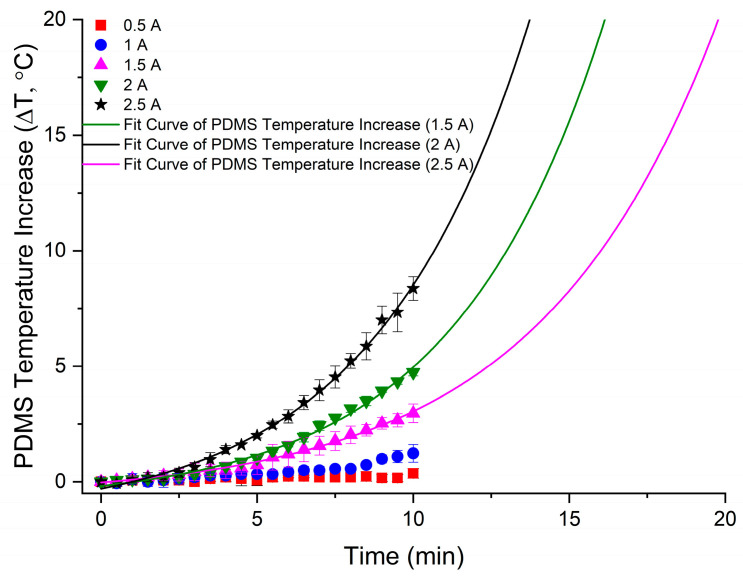
Temperature increase on the surface of a microfluidic device placed between the coils at different currents applied for a period of 10 min. The plot has been normalised to account for a difference in room temperature. For 1.5 A, 2.0 A and 2.5 A, the temperature was extrapolated by fitting the data to an exponential model. Error bars indicate standard deviation (*n* = 3).

The duration of operation and the current through the coils are factors that lead to Joule heating in the wires. This heat can be transferred to the surroundings which could affect the temperature experienced by MTB in the microfluidic device. Recent studies have shown that the average swimming speed of MTB does not significantly change when the temperature is increased from 25 °C to 37 °C, but it does increase significantly when the temperature is increased to 43 °C [20]. Therefore, it is necessary for any temperature increase at the microfluidic device to be less than 18 °C, and ideally less than 12 °C, above room temperature. Figure 5 presents the measured temperature difference of the surface of the microfluidic device over the course of 10 min for different applied electric currents. For lower currents corresponding to a magnetic field of 1.5 to 3 mT, the change in temperature at the upper surface of the microfluidic device is less than 1.5 °C. This temperature increase is insignificant and can be ignored as a potential bias for magnetotaxis studies. The highest increase in temperature at the microfluidic device was 8.4 ± 0.5 °C after 10 min with the current at 2.5 A, corresponding to a 7.8 mT magnetic field. Still, an increase of 8.4 ± 0.5 °C will not result in any alteration of the magnetotactic behaviour of MTB, according to previous studies [20]. Therefore, the platform presented in this work can be effectively used for magnetotaxis research without perturbing the magnetotactic bacterial behaviour due to heating, while supplying linear magnetic fields that override the geomagnetic field.

Table 1 shows the exponential regressions for 1.5, 2.0 and 2.5 A. The regressions are used to extrapolate the duration at each current when the temperature exceeds 18 °C above room temperature, occurring at 19.20 min, 15.15 min and 13.25 min, respectively. Therefore, the temperature increase will not affect the swimming speeds of MTB during experiments using the microfluidic–electromagnetic platform for durations shorter than the aforementioned times. Considering that the MTB motility observations and data acquisition require short periods of time, typically less than 1 min [11,26], the platform can be effectively used for MTB studies without significantly influencing the MTB swimming behaviour. Still, for lower currents that produce a strong enough electromagnetic field to override the geomagnetic field, the generated heat results in increases below 1.5 °C.

**Figure 6:**
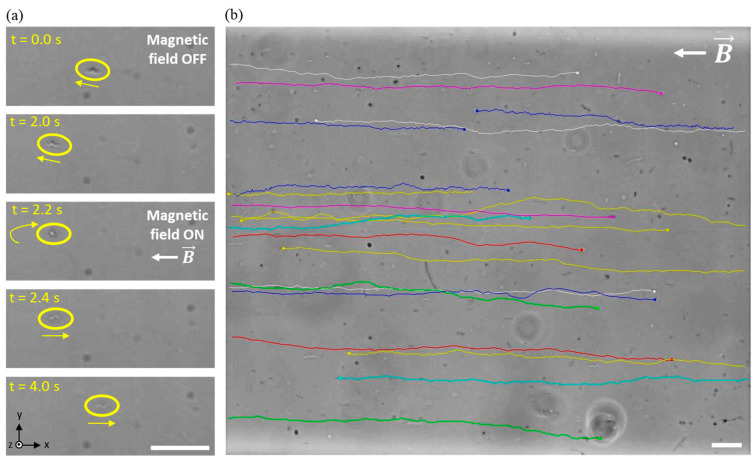
(**a**) A frame-by-frame sequence of an MTB cell turning to align with a 4.0 mT field. (**b**) Tracking overlay of 20 MTB swimming in a 4.0 mT horizontal magnetic field. Scale bars = 20 μm.

Using the FEA results, the platform was redesigned and the final version fabricated using the methodology described in the Section 2. Then, the effectiveness of the platform for the study of individual MTB, under directed magnetotactic navigation, was tested experimentally. Figure 6 presents the response of MTB to a 4.0 mT magnetic field. The results, collectively, verify the linear magnetotactic navigation within the microfluidic–electromagnetic coils platform. Figure 6a shows an image sequence of a bacterium turning in the z-direction. This behaviour, occurring instantaneously, confirms the presence of magnetosomes in the cell and consequential passive alignment with the magnetic field lines. Once aligned with the field, the motile MTB continue to swim along the field lines, as shown in Figure 6b. Although the experiments in this work include only linear directed motion, Codutti et al. have studied the curvilinear motion that revealed details on the U-turn radii of the trajectories of MTB while swimming inside circular microtraps, under the influence of a magnetic field produced by electromagnetic coils [31].

The MTB navigated in both directions along the field lines, exhibiting axial magnetotaxis which is expected in an axenic culture [32]. Nonmotile cells could be seen only rotating on the spot to align with the field, with no axial translation, whereas the trajectories of the motile cells were redirected in alignment with the magnetic field. These results demonstrate the effectiveness of the microfluidic–electromagnetic coils platform for successfully directing MTB, via magnetotaxis, in a microfluidic environment.

**Figure 7:**
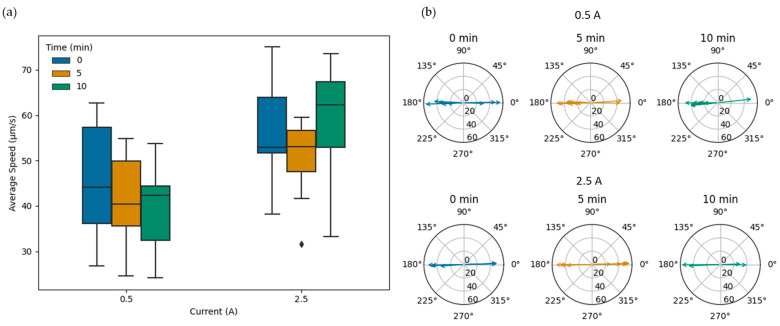
MTB velocities under the influence of applied electromagnetic fields at 0.5 and 2.5 A. (**a**) Box plot of the average velocity of the bacteria at 0, 5 and 10 min for the two different currents, showcasing the median and limits based on the quartiles (*n* = 10). The diamond represents a single data point for average speed at 2.5 A. (**b**) Radial plots of the average angles of the MTB swimming trajectories (initial and final points).

An additional experiment was conducted to investigate the effect of the temperature increase, as a result of the applied electromagnetic field, on the speed of MTB in directed magnetotactic motion. The bacteria were placed in the microfluidic chip, and the electromagnetic field was applied (t = 0) for 10 min, at two different currents, 0.5 and 2.5 A, corresponding to the current limits used in the heating measurement experiment. Videos and image sequences of the bacteria were acquired at 0, 5 and 10 min, with 10 replicates, corresponding to a temperature increase of less than 1 °C for the 0.5 A current at all times and 2.5 °C at 5 min and 8.4 °C at 10 min for the 2.5 A current. The speeds and trajectory angles, shown in Figure 7, were estimated using a custom Python code from the tracked positions and timestamps from the ImageJ-processed sequences. The average speeds at 0, 5 and 10 min were, correspondingly, 45.6 ± 12.38, 41.1 ± 10.11 and 40.2 ± 9.60 µm/s for the 0.5 A current and 56.4 ± 10.59, 50.6 ± 8.61 and 58.9 ± 13.03 µm/s for the 2.5 A current. The box plots in Figure 1a provide additional information on the speeds of the MTB at the different times and applied currents. The results suggest that the influence of the heating from the electromagnetic coils on the bacterial speed is not significant (*p* = 0.31), in agreement with previous studies [20]. The trajectories of the bacteria, in all cases, were well aligned with the magnetic field lines, as shown in Figure 7b. Only a couple of bacteria exhibited a slight deviation of about 5 degrees or less.

## 4. Conclusions

A customised microfluidic–electromagnetic coils platform was developed to study the magnetotaxis behaviour of MTB under precise and controlled magnetic fields at single-cell levels. The platform can be easily incorporated into a microscope stage, enabling real-time data acquisition of MTB magnetotactic behaviour, supporting microfluidic devices on standard microscope slides. The coils provide a tunable, linear magnetic field between 1 and 10 mT, suitable for directing MTB within a volume of 216 mm^3^. The capability of the platform for applying uniform electromagnetic fields across the microfluidic chip and for simultaneously studying the magnetotactic behaviour of individual microswimmers was demonstrated experimentally by effectively directing bacteria along the field lines at 4.0 mT while confined in a 50 μm deep, 300 μm wide microfluidic channel. The temperature changes at the microfluidic device were measured to ensure that the potential heating from the coils does not affect the motility characteristics of MTB nor the environmental properties of the medium. At the lowest currents used in the experiments, the platform can effectively produce a strong enough electromagnetic field to override the geomagnetic field, with a temperature increase less than 1.5 °C. At the highest current of 2.5 A, corresponding to a magnetic field of 7.8 mT, significant heating, enough to alter the magnetotactic behaviour of MTB, occurs after 13.25 min, suggesting that the platform can be safely used for typical magnetotactic studies with duration of less than 10 min. Still, the experimental results in the study revealed that the average magnetotactic speeds of the bacteria are not influenced by the heating generated by the coils. In addition, the design of the platform allows for the integration of cooling systems that could facilitate longer experiments at these higher currents.

## Figures and Tables

**Table 1:** Exponential regression results for the temperature increase at the surface of the microfluidic device over time with currents of 1.5 A, 2.0 A and 2.5 A.

Equation	y = A1*exp(x/t1) + y0		
Plot	PDMS Temperature Increase (1.5 A)	PDMS Temperature Increase (2 A)	PDMS Temperature Increase (2.5 A)
y0	−0.68 ± 0.11	−0.93 ± 0.21	−1.6 ± 0.23
A1	0.65 ± 0.11	0.75 ± 0.15	1.3 ± 0.16
t1	5.7 ± 0.47	4.8 ± 0.40	4.9 ± 0.26
R-Square (COD)	0.99	0.99	0.99
Adj. R-Square	0.99	0.99	0.99

## Data Availability

Not applicable.
